# Phenotypic characterization of a novel hAb‐KI^loxP^;hAPOE4;Trem2^R47H_NSS^ mouse model

**DOI:** 10.1002/alz.092579

**Published:** 2025-01-03

**Authors:** Angela Gomez‐Arboledas, Enikö Kramár, Shimako Kawauchi, Celia Da Cunha, Yueh Hao Lu, Giedre Milinkeviciute, Jonathan Neumann, Marcelo A Wood, Andrea J Tenner, Frank M LaFerla, Grant R MacGregor, Kim N. Green

**Affiliations:** ^1^ University of California, Irvine, Irvine, CA USA; ^2^ University of California Irvine, Irvine, CA USA

## Abstract

**Background:**

Genome‐Wide Association Studies (GWAS) identified ApoE4 and Trem2*R47H as two of the strongest genetic risk factors for late‐onset Alzheimer’s Disease (LOAD). As part of our efforts to develop mouse models that better recapitulate LOAD, at Model Organism Development & Evaluation for Late‐Onset Alzheimer’s Disease (MODEL‐AD) consortium at University of California – Irvine, we have created a triple homozygous mouse model that combines our previously developed hAb‐KI^loxP^ mice (Jackson Lab #031050), Trem2^R47H NSS^ (Jackson Lab #034036) and a humanized ApoE4 (Jackson Lab #027894), to evaluate the interactions between aging, hAPOE4, TREM2*R47H, and hAb.

**Method:**

By breeding the hAb‐KI^loxP^, hApoE4 and Trem2^R47H NSS^, we obtained triple homozygous (HO) mice and we then generated four different groups: WT (C57BL6/J), hAb‐KI^loxP^ HO, hAb‐KI^loxP^ HO;hApoE4 HO and hAb‐KI^loxP^ HO;hApoE4 HO;Trem2^R47H NSS^ HO. All four groups were then aged to 4, 12, 18 and 24 months of age, when coronal hippocampal slices were prepared, and long‐term potentiation recordings were obtained. Additionally, we also measured soluble and insoluble Ab40 and Ab42 as well as plasma neurofilament light chain (NfL).

**Result:**

hAb‐KI^loxP^ mice showed a significant reduction in mean potentiation 50‐60 minutes post TBS, when compared to WT mice at 4, 12, 18 and 24 months of age, indicative of an LTP deficit associated to human Ab. Interestingly, both hAPOE4 and Trem2^R47H NSS^ variants completely prevent the LTP deficits observed on the hAb‐KI^loxP^ mice from 4 months of age. In addition, cortical soluble Ab40, Ab42 and the Ab42/Ab40 ratio were significantly decreased in hAb‐KI^loxP^;hAPOE4 and hAb‐KI^loxP^;hAPOE4;Trem2^R47H NSS^ mice at 24 m of age, when compared to hAb‐KI^loxP^. However, plasma NfL levels were significantly increased in hAb‐KI^loxP^;hAPOE4;Trem2^R47H NSS^ mice at 24 months of age, indicative of axonal damage in this mouse model.

**Conclusion:**

hAb induces robust LTP deficits that are prevent by either hAPOE4 or TREM2*R47H, contrary to expectations. However, hAPOE4 and Trem2^R47H NSS^ variants have a cumulative detrimental effect on axonal damage when combined with aging.

